# Pre-Exposure to Volatile β-Ocimene Enhances Tomato Antiherbivore Resistance Against Polyphagous Lepidopteran Pest *Spodoptera litura* (Fabricius)

**DOI:** 10.3390/plants15193049

**Published:** 2026-10-06

**Authors:** Lu Tong, Yi Zhao, Hao Zhang, Yuanxin Ding, Ruofan Wu, Shiyao Xu, Qianrong Zhang, Xiaomin Song, Rensen Zeng, Yuanyuan Song, Yibin Lin, Xianhui Lin

**Affiliations:** 1 State Key Laboratory of Agricultural and Forestry Biosecurity, Key Laboratory of Ministry of Education for Genetics, Breeding and Multiple Utilization of Crops, College of Agriculture, Fujian Agriculture and Forestry University, Fuzhou 350002, China; tonglu1020@fafu.edu.cn (L.T.); zhaoyipgpr@163.com (Y.Z.); 15864667335@163.com (H.Z.); 15980196816@163.com (Y.D.); yuc8477@163.com (R.W.); xvshiyao2005@163.com (S.X.); rszeng@fafu.edu.cn (R.Z.); yyuansong@163.com (Y.S.); 2 Key Laboratory of Crop Biotechnology of Fujian Higher Education Institutes, Key Laboratory of Biological Breeding for Fujian and Taiwan Crops, Ministry of Agriculture and Rural Affairs, Fujian Agriculture and Forestry University, Fuzhou 350002, China; 3 Crops Research Institute, Fujian Academy of Agricultural Sciences, Fuzhou 350003, China; zhqrong@163.com; 4 State Key Laboratory of Plant Trait Design, CAS Center for Excellence in Molecular Plant Sciences, Shanghai Institute of Plant Physiology and Ecology, Chinese Academy of Sciences, Shanghai 200032, China; xmsong@cemps.ac.cn

**Keywords:** plant volatiles, anti-herbivore defenses, β-ocimene, defense priming, jasmonic acid, tomato

## Abstract

Herbivore-induced plant volatiles (HIPVs) are well recognized for their ability to recruit natural enemies of insect herbivores; however, their function in priming plant anti-herbivore defenses remains poorly elucidated. Here, we demonstrate that volatile exposure to a commercial β-ocimene isomer mixture (hereinafter referred to as β-ocimene) enhances anti-herbivore defense responses in tomato (*Solanum lycopersicum*) against the polyphagous lepidopteran pest *Spodoptera litura* (Fabricius). Larval infestation by *S. litura* significantly upregulated the expression of ten terpene synthase (*TPS*) genes that govern the biosynthesis of four dominant monoterpene HIPVs: β-ocimene, (-)-α-phellandrene, myrcene, and limonene. Preliminary bioassays revealed that pre-exposure to these four monoterpenes significantly enhances tomato resistance to *S. litura* larvae, with β-ocimene exhibiting the most pronounced effect. A 24 h pre-exposure to β-ocimene substantially augmented tomato antiherbivore resistance, and this protective response persisted for up to 10 days. Biochemical and molecular analyses revealed that pre-exposure to β-ocimene elevated the endogenous accumulation of jasmonic acid (JA) and jasmonoyl-isoleucine, along with increased transcript levels of the JA biosynthetic genes *LOXD* and *AOS*. Consistently, the JA biosynthesis-deficient mutant *spr8* failed to exhibit strengthened antiherbivore resistance upon β-ocimene pre-exposure. Collectively, our results demonstrate that β-ocimene pre-exposure enhances tomato antiherbivore resistance via activating JA biosynthesis, highlighting the potential ecological function of HIPVs in priming plant defense against insect pests.

## 1. Introduction

How sessile plants communicate with other organisms in the surroundings to adapt to various biotic and abiotic stresses is vital for plant survival in nature. One of the most important strategies is to release volatile organic compounds (VOCs), which act as early warning, repellent, lure, and alert signals through mixed odor messages [1,2]. These VOCs display the physiological status of the emitter plant and are available for perception by animals and other plants in the community. The emission of VOCs plays a key role in plant communication with other organisms in the environment, including herbivores, parasitoids, predators, neighboring plants, beneficial microorganisms, and pollinators [3,4,5]. Upon insect herbivory, plants release distinct blends of VOCs that are named herbivore-induced plant volatiles (HIPVs). HIPVs are usually triggered by mechanical wounding caused by insect feeding plus insect oral secretions. It has been well documented that HIPVs are able to impact the behavior of herbivores and natural enemies and act as chemical cues that induce or prime plant defenses [4,6,7]. These HIPVs can also serve as a warning signal to prime defenses in unwounded parts of the same plant and unwounded neighboring plants. HIPV-exposed plants then show physiological reprogramming and enhanced resistance to cumulative seasonal herbivory.

Terpenoids constitute the largest and most structurally diverse class of plant secondary metabolites and serve as core components of HIPVs, which mediate plant defensive responses against insect herbivory. HIPVs mainly comprise terpenes, green leaf volatiles (GLVs), aromatic compounds, and amino acid derivatives. A substantial fraction of the HIPVs are volatile terpenes, which are abundantly produced in plant response to stresses. These terpenes, comprising isoprene, monoterpenes, and sesquiterpenes, display diverse ecological functions. Their emission is highly specific to herbivore feeding stimuli rather than simple mechanical damage [8]. They can directly repel herbivores or indirectly protect the plant by attracting natural enemies of the herbivores. They can also prime neighboring plants for enhanced induction of defense response upon subsequent herbivore attack [9]. Upon perception, specific volatile terpenes can activate distinct signaling pathways in receiver plant cells, leading to provocation of plant defense responses. For instance, (*E*)-4,8-dimethyl-1,3,7-nonatriene released from the leaves of herbivore-infested sweet potato (*Ipomoea batatas*) plants systemically induces the production of sporamin protease inhibitor in neighboring healthy plants [10].

β-Ocimene, a core terpenoid volatile compound released by multiple crop species upon lepidopteran herbivory, has been frequently detected in the volatile blends of infested tomato, rice, Arabidopsis, tobacco, and tea (*Camellia sinensis*) plants [11,12,13,14]. Previous ecological studies have confirmed that β-ocimene participates in plant indirect defense by recruiting parasitoid wasps and predatory arthropods in agricultural ecosystems [15,16]. Beyond its ecological signaling functions, emerging studies suggest that specific terpene HIPVs can trigger defense priming in receiver plants, preparing them for faster and stronger defense responses upon future pest challenge [17,18]. Defense priming is a unique physiological state that differs from direct defense activation: primed plants do not exhibit constitutive defense activation and avoid unnecessary energy consumption under normal conditions, but display dramatically amplified defense responses once exposed to subsequent biotic stress [18,19]. This energy-efficient defense strategy plays a vital role in improving plant fitness under variable herbivory pressure [9]. However, whether β-ocimene serves as a functional priming signal to modulate tomato antiherbivore resistance, as well as the duration and molecular regulatory basis of such priming effects, has not been systematically elucidated [20].

The jasmonic acid (JA) signaling pathway is the core regulatory module governing plant defense responses against insect herbivores [21,22]. Upon insect feeding, plants rapidly accumulate JA and its bioactive conjugate jasmonoyl–isoleucine (JA-Ile), which further activates the expression of downstream defense-related genes and defensive metabolites to suppress herbivore growth and feeding [23,24]. Multiple studies have demonstrated that HIPV-induced defense priming is largely dependent on the JA signaling cascade in most plant species, especially solanaceous crops [10,25]. Genes involved in JA biosynthesis, such as *LOXD* and *AOS*, are key rate-limiting genes that determine endogenous JA accumulation and subsequent defense activation in tomato [21]. Mutants with defective JA biosynthesis typically exhibit severely impaired antiherbivore resistance and fail to respond to HIPV priming signals [26]. Despite the established link between HIPVs and JA signaling, it remains unknown whether β-ocimene-triggered tomato defense priming against *S. litura* is mediated by the JA pathway, and genetic evidence is urgently needed to verify this regulatory mechanism [13].

In this study, we employed tomato, one of the most economically valuable vegetable crops worldwide, and the polyphagous lepidopteran pest *Spodoptera litura*, a highly destructive herbivore species, as a research system to characterize β-ocimene-mediated priming of tomato antiherbivore resistance and assess the temporal persistence of such priming effects. Ten tomato terpene synthase (*TPS*) genes exhibited marked transcriptional upregulation upon infestation by *S. litura* larvae. We first screened five herbivore-responsive monoterpenes, including β-ocimene (a commercial isomer mixture: 69% *(E)*-β-ocimene and 29% *(Z)*-β-ocimene), (R)-(+)-limonene, (-)-α-phellandrene, myrcene, and linalool, for their ability to boost tomato resistance to *S. litura*, among which β-ocimene showed the strongest activity. Based on the screening outcomes, we focused subsequent mechanistic analyses on the β-ocimene isomer mixture. We systematically examined the physiological and molecular responses of β-ocimene-pretreated tomato plants by quantifying the accumulation of JA and JA-Ile, as well as the transcript abundance of key JA biosynthesis genes. Furthermore, the JA biosynthesis-deficient mutant *spr8* was utilized for genetic validation to verify whether β-ocimene-triggered defense priming is dependent on endogenous JA accumulation. Collectively, our findings establish β-ocimene as a critical terpene volatile that primes tomato defense against *S. litura* attack, uncover the core JA-dependent molecular mechanism underlying this volatile-mediated priming response, and advance the current understanding of HIPV-mediated plant–insect interactions.

## 2. Results

### 2.1. Insect Herbivory Upregulates the Expression of Terpene Synthase Genes in Tomato

**Figure 1:**
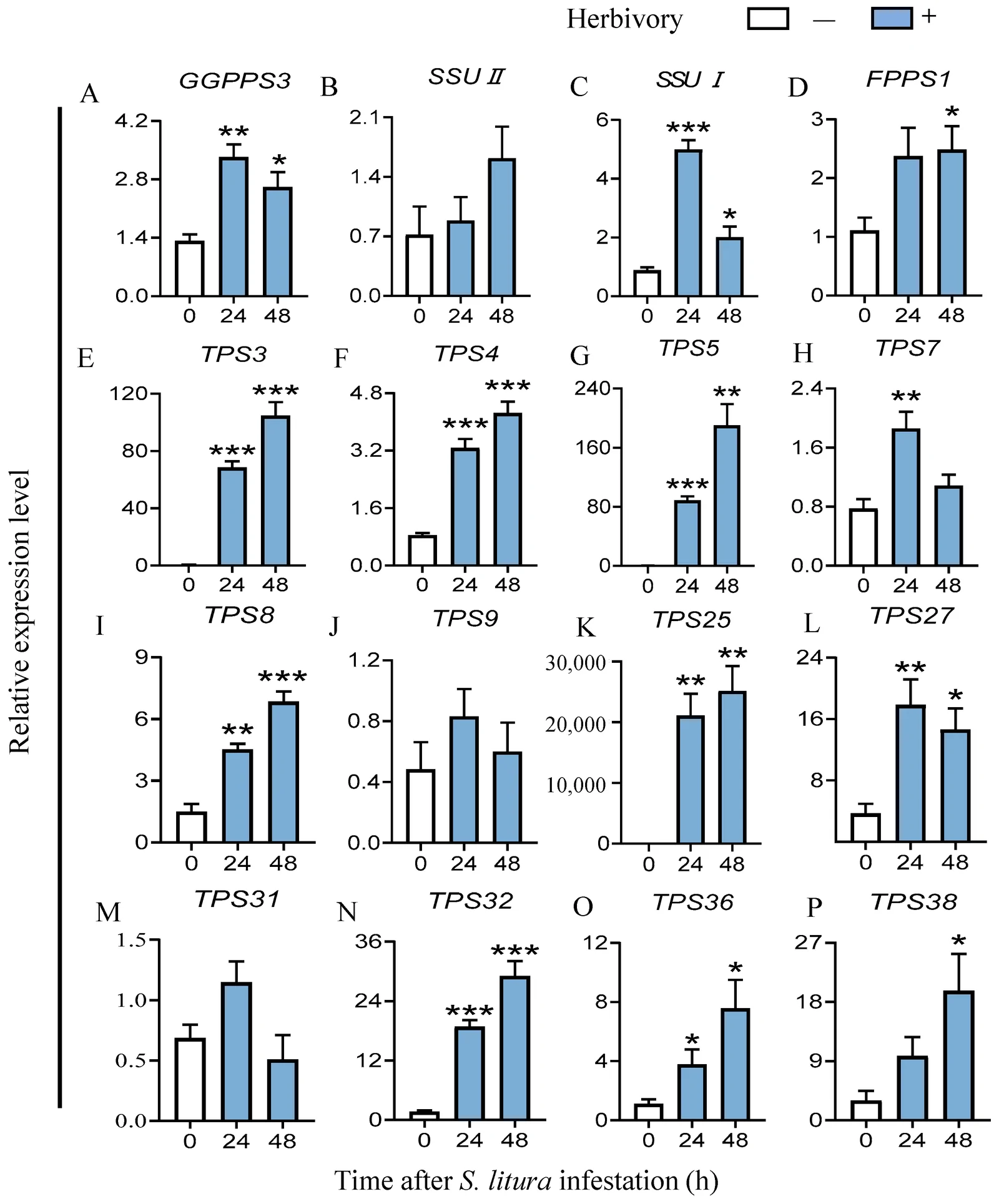
Transcription induction of terpene biosynthesis related genes in tomatoes by larval infestation of *Spodoptera litura.* Each five-leaf-stage tomato plant (cv. Moneymaker) was inoculated with five third-instar *S. litura* larvae. The leaves were sampled for RNA extraction and RT-qPCR 24 and 48 h after larval inoculation. Herbivory “−” and “+” indicate *S. litura* uninoculated and inoculated tomato plants, respectively. Data are mean ± SE (*n* = 3 plants). Asterisks indicate significant differences (* *p* < 0.05; ** *p* < 0.01; *** *p* < 0.001; Student’s *t*-test).

RT-qPCR analysis demonstrated that infestation by *Spodoptera litura* larvae significantly induced the expression of numerous tomato TPS genes at 24 h and 48 h post-feeding (Figure 1A–P). Ten TPS genes, including *TPS3*, *TPS4*, *TPS5*, *TPS7*, *TPS8*, *TPS25*, *TPS27*, *TPS32*, *TPS36*, and *TPS38*, exhibited significant transcriptional upregulation upon larval infestation.

Among these induced genes, *TPS3*, *TPS4*, *TPS5*, *TPS7*, and *TPS25* belong to the geranyl diphosphate (GPP) biosynthetic branch, which encodes monoterpene synthases responsible for the production of camphene, α-phellandrene, linalool, myrcene, limonene, and β-ocimene. The transcript levels of these genes were markedly elevated at 24 h, 48 h, or both time points after *S. litura* infestation. Additionally, insect herbivory significantly upregulated *SSUI*, which encodes the rate-limiting enzyme governing GPP-branch terpene biosynthesis. Moreover, *S. litura* infestation also activated the transcription of TPS genes involved in the farnesyl diphosphate (FPP) biosynthetic branch. Specifically, the sesquiterpene synthase genes *FPPS1*, *TPS9*, *TPS27*, *TPS31*, *TPS32*, *TPS36*, and *TPS38* were differentially upregulated following larval feeding (Figure 1J,L,M–P).

### 2.2. Terpene Volatiles Prime Tomato Antiherbivore Defenses Against S. litura

**Figure 2:**
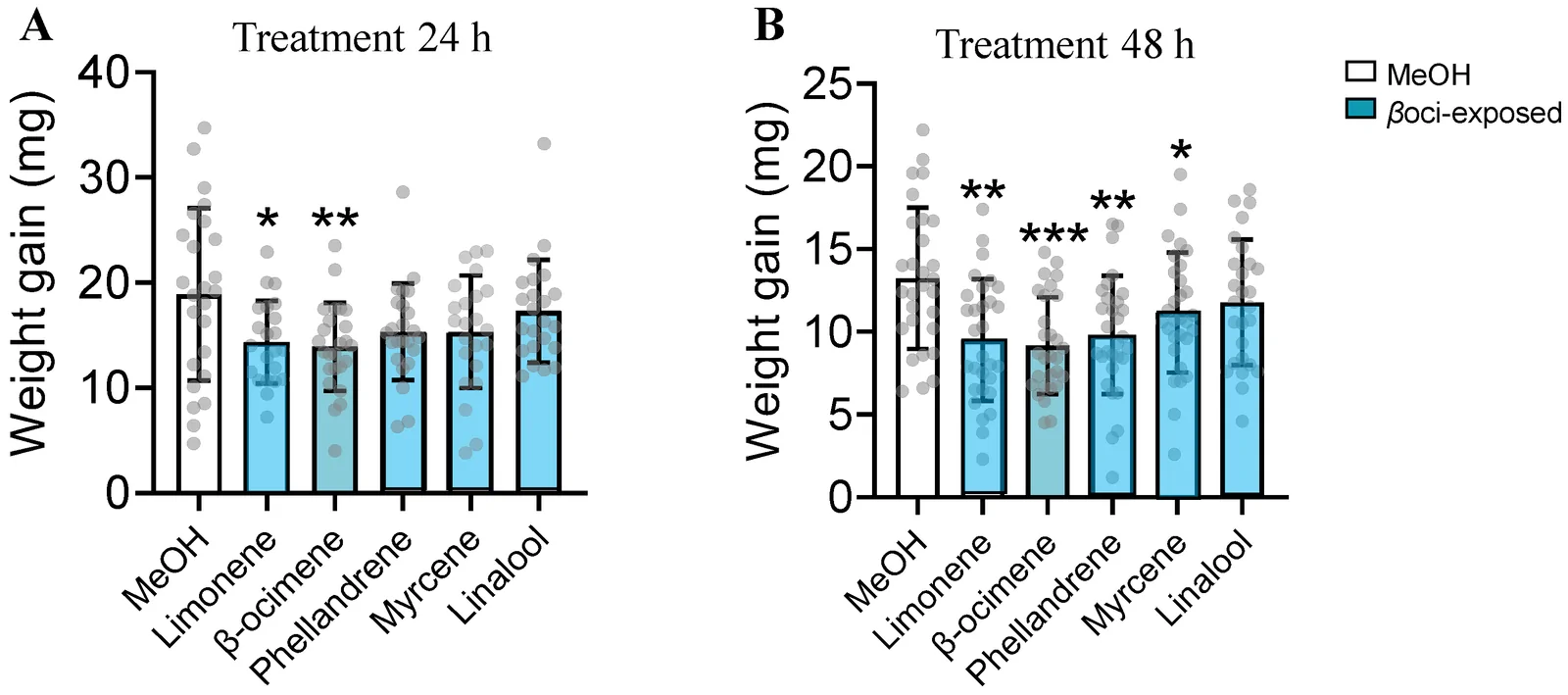
Pre-exposure to individual commercial terpene volatiles enhances antiherbivore resistance in tomato plants. Tomato plants (cv. Moneymaker) were pre-exposed to *(R)*-(+)-limonene, *β*-ocimene, *α*-phellandrene, myrcene, and linalool for 24 h (**A**) and 48 h (**B**), followed by feeding with five third-instar *S. litura* larvae. Tomato plants exposed to the solvent MeOH served as the control. Weight gain was obtained 24 h after feeding on the pre-exposed plants. Data are mean ± SD ((**A**) *n* = 22–25 surviving larvae; (**B**) *n* = 27–30 surviving larvae). Asterisks indicate significant differences between the control (unexposed) and exposed groups (* *p* < 0.05; ** *p* < 0.01; *** *p* < 0.001; Student’s *t*-test).

Five commercially obtained terpene preparations, including a commercial β-ocimene isomer mixture (≥98%, mixture of isomers: 69% *(E)*-β-ocimene and 29% *(Z)*-β-ocimene), (R)-(+)-limonene, (-)-α-phellandrene, myrcene, and linalool, were included as an exploratory panel based on previously characterized products of herbivory-responsive tomato TPS enzymes and the related literature [27]. At 24 h post-feeding, pre-exposure to limonene, β-ocimene, and α-phellandrene reduced larval weight gain by 24% (*p* = 0.022), 26% (*p* = 0.009), and 19% (*p* = 0.068), respectively (Figure 2A). At 48 h post-feeding, the inhibitory effects were enhanced: limonene, β-ocimene, (-)-α-phellandrene, and myrcene pre-exposure decreased larval weight gain by 29% (*p* = 0.001), 31% (*p* < 0.001), 23% (*p* = 0.007), and 17% (*p* = 0.042), respectively, while linalool showed no significant effect (13% reduction, *p* = 0.159) (Figure 2B). Collectively, these results demonstrate that β-ocimene acts as the most effective terpene volatile priming tomato antiherbivore resistance in tomato.

### 2.3. β-Ocimene Exposure Confers Long-Lasting Antiherbivore Defense Priming in Tomato

**Figure 3:**
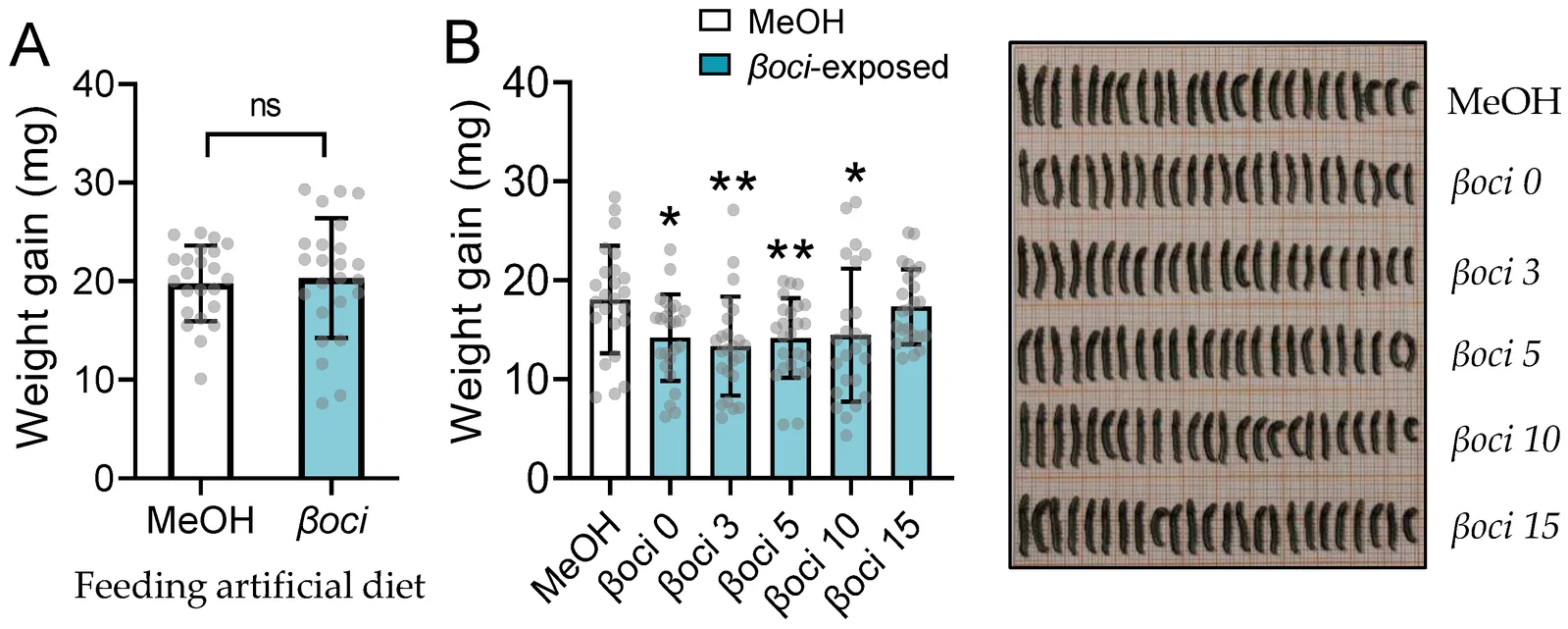
Pre-exposure to *β*-ocimene primes persistent antiherbivore resistance in tomato plants. (**A**) Weight gain of *S. litura* larvae fed on artificial diets with or without *β*-ocimene (*βoci*) for 24 h. (**B**) Twenty-four-hour weight gain of *S. litura* larvae fed on tomato plants (cv. Moneymaker) exposed to *β*-ocimene for 24 h and then grown for 0, 3, 5, 10, and 15 d before larval inoculation. Data are mean ± SD (*n* = 24). Asterisks indicate significant differences between control (MeOH) and *βoci*-exposed treatments (ns, *p* > 0.05; * *p* < 0.05; ** *p* < 0.01; Student’s *t*-test).

Compared with the methanol control group, pre-exposure to the commercial β-ocimene isomer mixture significantly reduced larval weight gain of *S. litura* fed on tomato plants (cv. Moneymaker) by 21% (*p* = 0.01), 26% (*p* = 0.003), 22% (*p* = 0.007), and 20% (*p* = 0.01) at 0, 3, 5, and 10 days post-treatment, respectively. No significant inhibitory effect was observed at 15 days post-exposure (*p* = 0.599) (Figure 3A). These results indicate that β-ocimene-triggered antiherbivore defense persists for up to 10 days but diminishes within 15 days under the specified laboratory conditions.

No significant difference in larval weight gain was detected between the β-ocimene-treated and control larvae fed on artificial diets (*p* = 0.73) (Figure 3A), verifying that β-ocimene functions by priming plant endogenous defenses rather than directly affecting larval growth.

### 2.4. Effect of Cultivars on β-Ocimene-Mediated Defense Priming in Tomato

**Figure 4:**
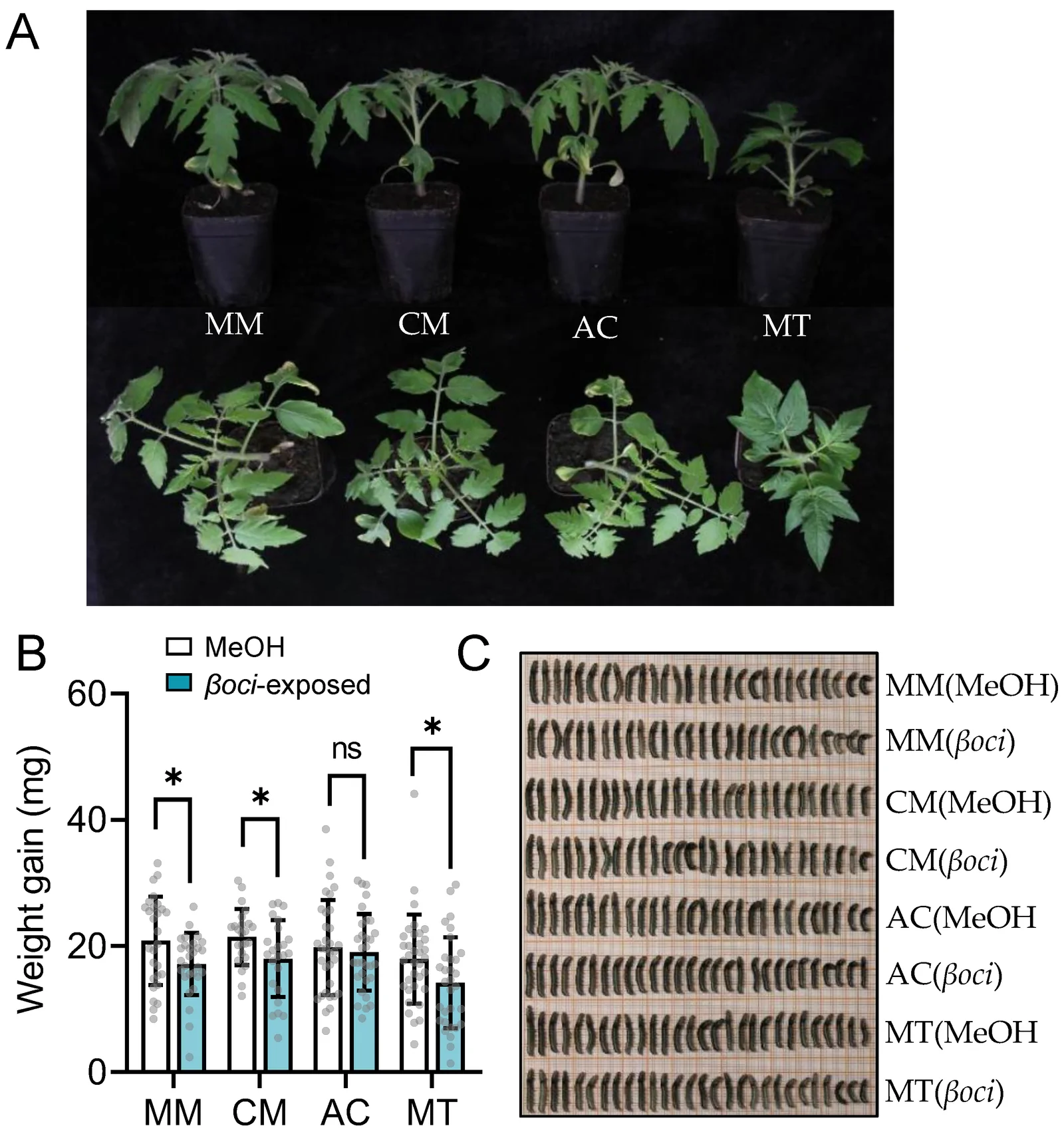
Weight gain of *Spodoptera litura* larvae fed on tomato plants of four cultivars pre-exposed to *β*-ocimene (*βoci*). The four cultivars were Castlemart (CM), Ailsa Craig (AC), Moneymaker (MM), and Micro-Tom (MT). (**A**) Growth phenotypes of four tomato cultivars. Weight gain (**B**,**C**) phenotypes of *S. litura* larvae fed on tomato plants of four tomato cultivars pre-exposed to *β*-ocimene for 24 h. Tomato plants pre-exposed to the solvent MeOH served as control. Data are mean ± SD (*n* = 24–31 surviving larvae). Asterisks indicate significant differences between control (MeOH) and *βoci*-exposed treatments (ns, *p* > 0.05; * *p* < 0.05; Student’s *t*-test).

Tomato plants of four cultivars, including Moneymaker (MM), Castlemart (CM), Ailsa Craig (AC), and Micro-Tom (MT), at comparable developmental stages were exposed to the commercial β-ocimene isomer mixture for 24 h and then used in larval-feeding assays (Figure 4A). Significant enhancement of antiherbivore resistance was observed in the MM, CM, and Micro-Tom cultivars. Specifically, larval weight gain was reduced by 18% (*p* = 0.026), 16% (*p* = 0.03), and 21% (*p* = 0.037) in β-ocimene-pre-exposed MM, CM, and Micro-Tom plants relative to their corresponding solvent controls, respectively (Figure 4B,C). In contrast, the AC cultivar exhibited no significant priming response (*p* = 0.662).

### 2.5. β-Ocimene-Induced Defense Priming Requires Intact, Living Tomato Plants

**Figure 5:**
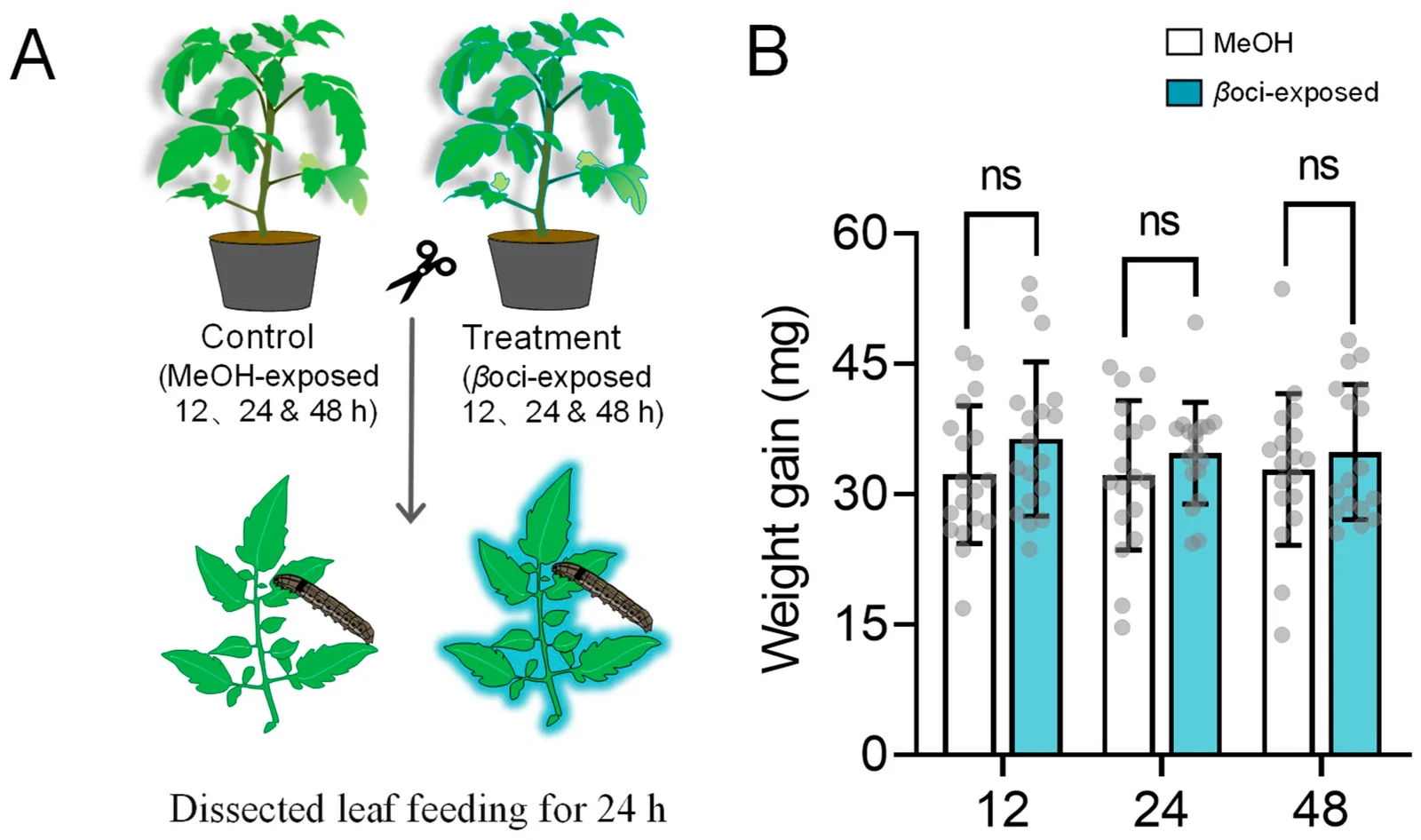
Performance of *Spodoptera litura* larvae fed on dissected leaves of tomato plants with and without exposure to *β*-ocimene (*βoci*). Tomato plants (cv. Micro-Tom) were exposed to *βoci* for 12, 24, and 48 h. The solvent (MeOH) exposure served as control. Then the leaves were dissected with scissors and inoculated with three third-instar larvae. (**A**) Schematic diagram of experimental design. (**B**) Weight gain of larvae fed on *βoci*-exposed and unexposed dissected leaves for 24 h. Data are mean ± SD (*n* = 18 surviving larvae per comparison). The ‘ns’ indicates no significant difference between exposed and unexposed treatments (*p* > 0.05; Student’s *t*-test).

Tomato plants (cv. Micro-Tom) were exposed to β-ocimene for 12, 24, or 48 h, after which fully expanded leaves were detached and used for 24 h *S. litura* feeding assays (Figure 5A). Relative to the respective methanol controls, larval weight gain increased by 13%, 8%, and 6% after the three exposure periods (*p* = 0.152, 0.304, and 0.479, respectively; Figure 5B). No significant differences in larval weight gain were detected between larvae fed on β-ocimene pre-exposed detached leaves and those fed on control detached leaves (Figure 5B). This finding indicates that β-ocimene-mediated defense priming is exclusively functional in intact, living tomato plants and cannot be sustained in isolated leaf tissues.

### 2.6. β-Ocimene-Elicited Antiherbivore Priming Is Dependent on JA Biosynthesis

**Figure 6:**
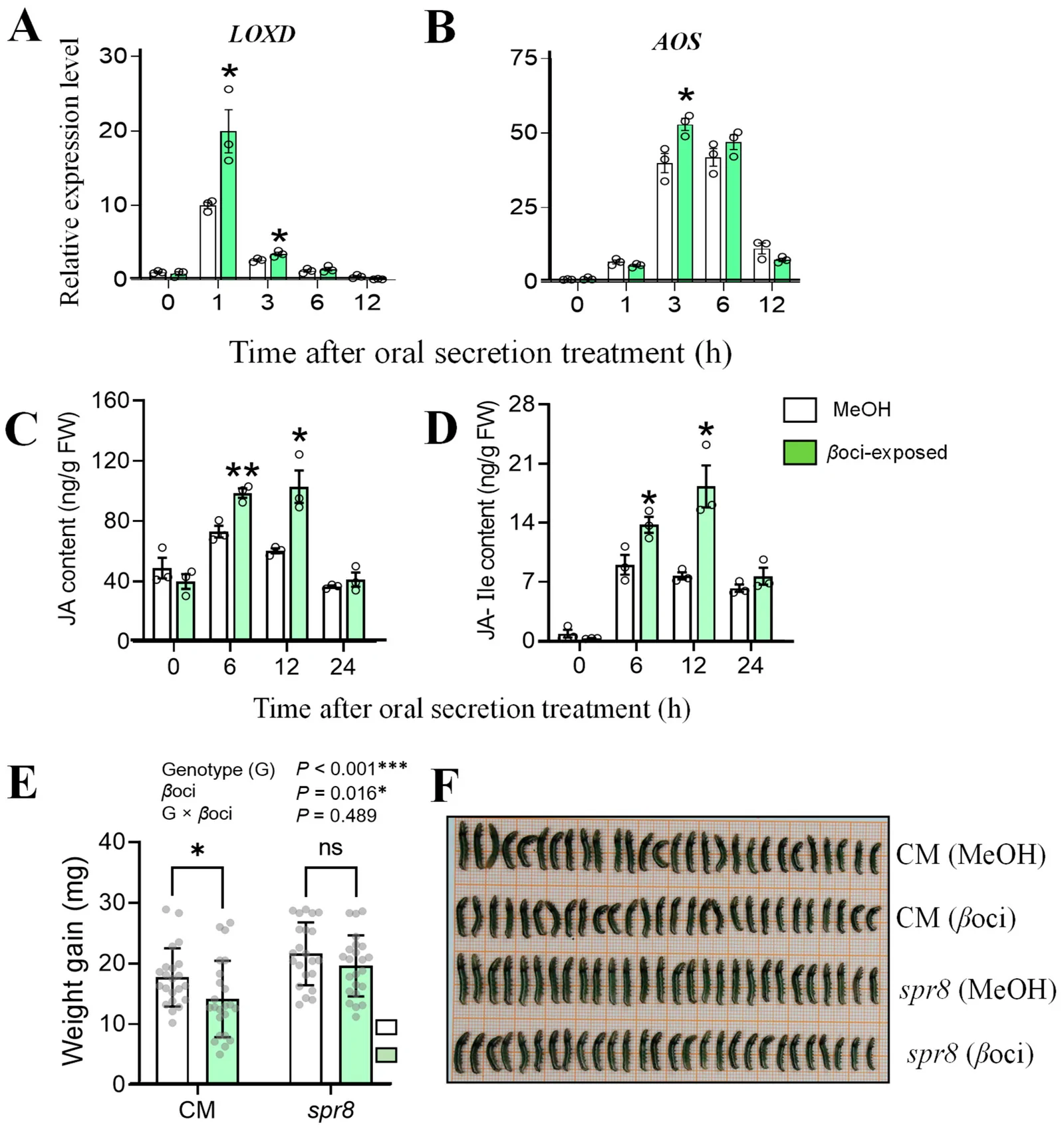
The jasmonic acid (JA) signaling pathway is involved in *β*-ocimene-mediated tomato defense against *Spodoptera litura.* Tomato plants (cv. Castlemart) were exposed to the commercial β-ocimene isomer mixture (*βoci*) for 24 h. Then the plants were treated with wounding plus oral secretion of *S. litura* (WOS). At 1, 3, 6, 12, and 24 h after WOS treatment, the plant leaves were sampled for RNA extraction and jasmonic acid (JA) and jasmonoyl–isoleucine (JA-Ile) quantification. Samples at 0 h were collected before WOS. (**A**–**D**) Transcript levels of JA biosynthesis genes *LOXD* (**A**) and *AOS* (**B**) and contents of JA (**C**) and JA-Ile (**D**) in the leaves of *βoci*-exposed tomato plants at different time points after WOS treatments. Weight gain (**E**,**F**) phenotypes of *S. litura* larvae fed on tomato plants of wild-type CM and JA-deficient mutant *spr8* exposed to *β*-ocimene for 24 h. Data are mean ± SE (*n* = 3 biological replicates for (**A**–**D**)) and mean ± SD (*n* = 28–30 surviving larvae for (**E**,**F**)). Asterisks indicate significant differences between control (MeOH) and *βoci*-exposed treatments (ns, *p* > 0.05; * *p* < 0.05; ** *p* < 0.01; *** *p* < 0.001; Student’s *t*-test).

Tomato plants (cv. Castlemart) were pre-exposed to methanol or a commercial β-ocimene isomer mixture for 24 h before WOS treatment. At 0 h (immediately after pre-exposure and prior to WOS exposure), transcriptional levels of *LOXD* and AOS and contents of JA and JA-Ile showed no significant differences between the methanol control and β-ocimene-pretreated plants. However, upon WOS stimulation, β-ocimene-pre-exposed plants exhibited significantly higher accumulation of JA and JA-Ile at 6 h and 12 h post-treatment (Figure 6C,D). Meanwhile, the transcriptional levels of *LOXD* and *AOS* were markedly upregulated at 3 h post-WOS treatment in β-ocimene-primed plants (Figure 6A,B).

Subsequent insect bioassays showed that β-ocimene pre-exposure significantly suppressed *S. litura* larval weight gain in wild-type plants (cv. Castlemart) (*p* = 0.039). In contrast, the priming effect was completely abolished in *spr8* mutant plants, with no significant difference in larval growth between β-ocimene-exposed and control mutants (*p* = 0.198) (Figure 6E,F). Collectively, these results demonstrate that intact JA biosynthesis is involved in β-ocimene-triggered antiherbivore defense priming in tomato.

## 3. Discussion

Herbivore-induced plant volatiles (HIPVs) constitute essential airborne signaling molecules that modulate plant–insect interactions and prime plant defense responses [28]. Nevertheless, the specific biological functions and molecular regulatory mechanisms of individual terpene-based HIPVs in tomato anti-herbivore resistance remain largely unclear. In this study, we investigated the effects of pre-exposure to five typical terpene volatiles (a commercial β-ocimene isomer mixture, myrcene, (-)-α-phellandrene, *(R)*-(+)-limonene, and linalool) on tomato resistance against the polyphagous herbivore *Spodoptera litura*. Our results further identified the β-ocimene isomer mixture as a key defensive priming signal that markedly enhances tomato resistance to *S. litura*. A 24 h pre-exposure to the β-ocimene isomer mixture induced a long-term defense priming effect in tomato, which persisted for up to 10 days and continuously strengthened plant anti-herbivore capacity.

Molecular assays further verified that β-ocimene-triggered defense priming is dependent on the activation of the jasmonic acid (JA) signaling cascade. Specifically, β-ocimene treatment significantly increased the accumulation of JA and jasmonoyl–isoleucine (JA-Ile) and upregulated the transcript levels of JA biosynthetic genes *LOXD* and *AOS* in wild-type tomato plants. Consistently, the JA biosynthesis-deficient tomato mutant *spr8* failed to exhibit β-ocimene-induced defense priming, confirming the important role of JA signaling in this regulatory process. Collectively, these findings elucidate the ecological function of β-ocimene in crop anti-herbivore defense and uncover a conserved JA-dependent regulatory module that underpins HIPV-mediated induced resistance in tomato.

Notably, our current bioassays did not distinguish the specific active β-ocimene isomer responsible for observed defense induction. In this study, we used a natural mixture of (E)-β-ocimene and (Z)-β-ocimene and focused on the overall biological function of total β-ocimene, rather than distinguishing the independent role of individual isomers. We have not claimed a specific function for any single ocimene isomer. Considering the extremely high cost of pure, single β-ocimene isomers, the verified antiherbivore priming activity of the commercially available β-ocimene isomer mixture also endows our findings with greater practical value and application potential for sustainable crop protection in agricultural production systems. Multiple previous studies have confirmed that tomato plants predominantly emit (*E*)-β-ocimene in response to insect herbivory [29,30], implying that (*E*)-β-ocimene may be the functional isomer driving the defensive priming observed in our study.

In addition, this study did not quantitatively detect the exact gaseous concentration of β-ocimene within the exposure containers. However, to rule out the potential interference of residual β-ocimene chemical toxicity and direct insecticidal effects on larval feeding performance, two independent control experiments were designed for verification. First, in the artificial diet exposure experiment, larvae were continuously exposed to the same β-ocimene treatment dosage as in the plant exposure assay. No significant inhibitory or adverse effects on larval feeding behavior and growth were observed (Figure 3A), which excludes the direct toxic effect of β-ocimene on *S. litura* larvae. Second, we performed independent feeding assays using detached leaves from β-ocimene-pre-exposed tomato plants. Consistent with the above results, no adverse larval feeding performance was detected, further confirming that residual β-ocimene chemicals on plant tissues did not interfere with insect growth. These two experiments collectively verify that the enhanced plant antiherbivore resistance originates from β-ocimene-induced plant defense priming, rather than residual chemical toxicity. Furthermore, previously published research by Ghosh and Venkatesan, as well as Luo et al., has demonstrated that β-ocimene exerts no significant inhibitory effects on the growth of *S. litura* larvae [31,32].

Intriguingly, previous evidence has revealed that exposure to (*E*)-β-ocimene elevates plasmatocyte abundance and phenol oxidase activity in *S. litura* larvae, which enhances the cellular immunity of the herbivore [32]. These findings collectively indicate that the suppressed larval growth of *S. litura* observed in our study is not caused by the direct toxicity of β-ocimene to larvae but primarily results from β-ocimene-mediated activation of endogenous anti-herbivore defense responses in tomato plants.

Defense priming is a unique and energy-efficient plant defense strategy that enables plants to rapidly and robustly activate defensive responses upon subsequent biotic stress, without constitutively activating defense pathways under normal growth conditions [18,19]. Consistent with the typical characteristics of plant defense priming, no significant differences in the levels of JA, JA-Ile, and the transcripts of JA biosynthetic genes (*LOXD* and *AOS*) were detected immediately after β-ocimene exposure (0 h; Figure 6A–D). However, upon subsequent *S. litura* feeding stress (WOS treatment), β-ocimene-pre-exposed tomato plants exhibited significantly enhanced accumulation of JA and JA-Ile, as well as markedly upregulated expression of *LOXD* and *AOS* (Figure 6A–F). This amplified defense response upon subsequent herbivore attack strongly confirms the priming effect of β-ocimene. Furthermore, our bioassays verified that 24 h pre-exposure to the β-ocimene isomer mixture induced a long-term defense priming effect in tomato plants, which persisted for up to 10 days and continuously enhanced plant resistance against herbivory (Figure 3B). Collectively, these physiological, molecular, and phenotypic results solidly demonstrate that β-ocimene pre-exposure primes and enhances tomato antiherbivore resistance. Previous studies have verified that multiple terpene volatiles participate in plant antiherbivore and anti-pathogen defense priming across diverse plant species [9,33]. β-Ocimene, as a ubiquitous monoterpene HIPV in terrestrial plants, has been reported to modulate defense responses in *Arabidopsis*, tobacco, and legume plants against lepidopteran pests and aphids [15,20,34,35]. Consistent with these previous findings, our results in tomato further expand the defensive spectrum of β-ocimene, confirming that this volatile can serve as an early warning signal to prime plant resistance against the destructive polyphagous pest *S. litura*. Notably, the 10-day persistent priming effect observed in this study highlights the practical superiority of β-ocimene as a long-lasting defensive elicitor, which differs from transiently induced direct defenses. This prolonged priming capacity is ecologically adaptive for field-grown tomato plants, as it allows crops to maintain a vigilant defensive state during the peak occurrence period of *S. litura*, effectively reducing continuous herbivore damage without incurring excessive growth–defense tradeoff costs [34,36].

JA is the core signaling phytohormone that governs plant direct and indirect antiherbivore defenses, and the activation of JA biosynthesis and signaling pathways is essential for resisting chewing insect pests such as *S. litura* [37]. Our results provide multiple lines of genetic and biochemical evidence demonstrating that β-ocimene-triggered defense priming is associated with stronger JA-related responses. First, exogenous β-ocimene pre-exposure significantly induced the accumulation of JA and its bioactive conjugate JA-Ile, accompanied by the upregulation of two key JA biosynthetic genes, *LOXD* and *AOS*, indicating that volatile β-ocimene exposure transcriptionally activates JA biosynthesis in tomato. Second, β-ocimene-provoked antiherbivore resistance was not detected in the *spr8* mutant, which is defective in endogenous JA synthesis, under the conditions tested, confirming the key role of JA signaling in this priming process. These results align with previous studies on HIPV-mediated defense priming, wherein terpene volatiles commonly prime plant defenses by potentiating JA signaling responsiveness [35,38].

Tomato employs a complex terpene volatile blend rather than a single compound to respond to herbivore infestation [39]. While the current study focused on the priming function of β-ocimene, the defensive roles of myrcene, phellandrene, limonene, and linalool remain to be explored. These co-induced terpenes may function synergistically with β-ocimene to enhance overall antiherbivore resistance or mediate distinct defensive strategies such as attracting natural enemies of *S. litura* (indirect defense) or repelling adult oviposition [40,41]. Future research is warranted to dissect the independent and synergistic functions of these terpene components, as well as their underlying signaling mechanisms, to fully elucidate the defensive network of tomato HIPVs against *S. litura*. In addition, this study only verified the indoor priming effect of β-ocimene. Field conditions involve complex environmental factors (e.g., temperature, humidity, mixed pest infestation, interplant volatile communication) that may affect the stability and efficiency of β-ocimene-mediated defense priming [34]. Field trials are therefore required to validate the practical application value of β-ocimene in crop pest management. The environmentally relevant exposure levels of β-ocimene and its ecological or agricultural significance require further study.

Despite the novel findings presented in this study, several limitations remain to be addressed. First, we focused exclusively on the JA signaling pathway, while other defense-related signaling pathways (e.g., salicylic acid, ethylene) may also participate in β-ocimene-regulated defense responses. The potential crosstalk between JA and other phytohormone pathways during β-ocimene priming remains unclear and requires further investigation. Second, the upstream molecular sensors and signal transduction components that perceive β-ocimene signals in tomato have not been identified. Emerging evidence indicates that β-ocimene may trigger chromatin remodeling and epigenetic modification to sustain long-term defense priming in plants [35], yet the specific receptor or regulatory proteins that recognize β-ocimene in tomato remain uncharacterized. Uncovering these upstream regulatory modules will help to fully resolve the molecular mechanism of HIPV signal transduction.

## 4. Materials and Methods

### 4.1. Plant Materials

Tomato (*Solanum lycopersicum*, cv. Moneymaker) seeds (accession no. LA2706) were obtained from the Tomato Genetics Resource Center (TGRC) at the Department of Plant Sciences of the University of California, USA. Multiple wild-type tomato cultivars, including Castlemart (CM), Ailsa Craig (AC), Moneymaker (MM), and Micro-Tom (MT), and the JA biosynthesis-deficient tomato mutant *spr8* (carrying a mutation in the *SlLoxD* gene), which was generated in the CM background [26], were generously provided by Professor Chuanyou Li from Shandong Agricultural University, China. MM was used for TPS expression, volatile candidate screening, and persistence experiments; MT was used for the detached leaf experiment; CM and *spr8* were used for the genetic comparison; and MM, CM, AC, and MT were used for the cultivar comparison.

All seeds were sterilized using a 10% H_2_O_2_ solution for 15 min and then rinsed with pure water 5–6 times. Sterilized seeds were uniformly sown in seedling pots filled with autoclaved moist substrate. After full expansion of two cotyledons, uniform seedlings were transplanted into individual plastic pots (6 cm diameter, 9 cm height) containing sterile substrate. Plants were cultivated in a programmable growth chamber under controlled conditions: a 16 h light/8 h dark photoperiod, day/night temperatures of 25 °C/20 °C, 70% relative humidity (RH), and a photosynthetic photon flux density (PPFD) of 300 μmol m^−2^ s^−1^. All seedlings were irrigated with Hoagland’s nutrient solution every three days. Plants with 4–6 fully expanded true leaves were selected for subsequent experimental treatments.

### 4.2. Insect Culture

Larvae of *Spodoptera litura* (Lepidoptera: Noctuidae) were originally collected from local maize fields in Fuzhou, Fujian Province, China. The insect colony was continuously reared on a standard artificial diet [42] for more than 40 consecutive generations in a constant-temperature insectary at Fujian Agriculture and Forestry University (Fuzhou, China). Rearing conditions were set at 25 ± 2 °C and 60% RH, with a 16 h light/8 h dark photoperiod.

### 4.3. Chemicals

The solvent and tested volatile compounds were methanol (HPLC grade; Aladdin; CAS 67-56-1), myrcene (≥98%; Macklin; CAS 123-35-3), *(R)*-(+)-limonene (≥98%; Sigma-Aldrich; CAS 5989-27-5), β-ocimene (≥98%, mixture of isomers; Macklin; CAS 13877-91-3; supplier-reported approximate composition 29% *(Z)*-β-ocimene and 69% *(E)*-β-ocimene), (-)-α-phellandrene (≥65%; Macklin; CAS 4221-98-1), and linalool (≥95%; Macklin; CAS 78-70-6).

### 4.4. Terpene Synthase Gene (TPS) Expression After Insect Herbivory

Previous biochemical characterization of the tomato TPS family showed that TPS3, TPS4, TPS5, TPS7, and TPS25 encode enzymes producing monoterpenes that include camphene, (-)-α-phellandrene, linalool, myrcene, limonene, and β-ocimene [27]. In particular, TPS25 uses geranyl diphosphate as a substrate and produces predominantly β-ocimene, including β-cis-ocimene and β-trans-ocimene [27]. For TPS-expression experiments, each five-leaf-stage MM plant was infested with five third-instar larvae for 24 or 48 h. Then the leaves were sampled for RNA extraction and qRT-PCR analysis.

### 4.5. Volatile Exposure and Insect Bioassays

To screen for defensive priming-active terpenes, five herbivory-induced terpene volatiles, namely, (*R*)-(+)-limonene, β-ocimene mixture of isomers, (-)-α-phellandrene, myrcene, and linalool, were selected for functional bioassays. Tomato seedlings (cv. Moneymaker) were pre-exposed to each terpene for 24 h and 48 h, respectively (Figure S1), followed by *S. litura* larval feeding assays, with methanol-exposed plants serving as the solvent control. Each transparent and sealed plastic container (40 cm × 40 cm × 40 cm) held four tomato plants. Each exposure treatment included three containers. Three larvae (initial body weight: 21–25 mg) that had been starved for 3 h were inoculated on the fully expanded functional leaves of each treated tomato plant. Body weight gain of surviving larvae was recorded after 24 h of feeding (Figure 2).

The volatile exposure procedure was modified from Ye et al. [43]. For volatile treatment, each transparent and sealed container (40 cm × 40 cm × 40 cm) was equipped with a 10 mL transparent glass diffuser containing 200 μL of treatment solution (20 μL pure β-ocimene mixture dissolved in 180 μL HPLC-grade methanol). A 10 mL amber glass vial (24 mm diameter × 50 mm height; bottle-mouth diameter 20 mm) was used as a diffuser. Each vial was closed with an open screw cap fitted with a polytetrafluoroethylene/rubber septum, which was pierced with a 1 μL micropipette tip. Control vials contained 180 μL methanol. Diffusers were prepared 1 h before the experiment, wrapped in aluminum foil. Tomato seedlings were continuously exposed to β-ocimene volatiles for 24 h, after which the diffusers were removed. For the control group, plants were exposed to an equivalent volume of pure methanol (180 μL) under identical conditions (Figure S1).

To exclude possible direct toxic or inhibitory effects of β-ocimene on *S. litura* larvae, third-instar larvae were reared on artificial diets supplemented with β-ocimene isomer mixture or solvent control (methanol, MeOH) in the same size of container described above [43]. Each treatment included three containers, and eight larvae were reared on artificial diets in each container. Larval weight gain was quantified after 24 h of feeding on β-ocimene-supplemented or unsupplemented control artificial diets (Figure 3A).

To characterize the duration of β-ocimene-induced defense priming, tomato seedlings were pre-exposed to a β-ocimene isomer mixture for 24 h, and larval feeding bioassays were conducted at 0, 3, 5, 10, and 15 days after diffuser removal. Uniform seedlings of tomato (cv. Moneymaker) were randomly divided into two experimental groups: a β-ocimene-exposed treatment group and a solvent control group, with four containers per group. For each exposure duration, each exposure container held two plants, and three third-instar *S. litura* larvae were placed on each seedling for the feeding bioassay. Plants were maintained in a bioassay room under consistent conditions (12 h light/12 h dark photoperiod, 21–25 °C, 70% RH, 250 μmol m^−2^ s^−1^ PPFD). Larval weight gain was measured after 24 h of feeding (Figure 3B).

To explore whether β-ocimene-mediated defense priming varies with genotype in tomato, four commonly used cultivars (Moneymaker [MM], Castlemart [CM], Ailsa Craig [AC], and Micro-Tom [MT]) at identical developmental stages were pre-exposed to a β-ocimene mixture or solvent methanol for 24 h prior to insect feeding bioassays. Each treatment included three containers, and each exposure container held four plants. Three third-instar *S. litura* larvae were placed on each plant. Body weight gain of surviving larvae was assessed after 24 h of feeding on β-ocimene pre-exposed seedlings of the four cultivars (Figure 4B).

To determine whether β-ocimene triggered antiherbivore priming functions in detached plant tissues, fully expanded leaves were harvested from Micro-Tom seedlings pre-exposed to β-ocimene for 12 h, 24 h, or 48 h. MeOH-treated plants served as the solvent control (Figure 5A). Each exposure container held two plants and each treatment included three containers. Three fully expanded leaves were dissected from each plant, and one third-instar *S. litura* larva was introduced onto each leaf. Larval weight gain was measured following 24 h of feeding on the leaves from β-ocimene-exposed and control plants.

### 4.6. Simulated Herbivory and JA Biosynthesis-Deficient Tomato Mutant spr8

To explore the regulatory role of the JA signaling pathway in β-ocimene-induced defense priming, a simulated herbivory treatment (wounding plus *S. litura* oral secretions, WOS) was applied. Oral secretions (OSs) of *S. litura* larvae were collected as previously reported [44]. Healthy fifth-instar *S. litura* larvae were gently held between fingers, and their oral cavities were stimulated with a 10 μL pipette tip to elicit secretion, yielding 10–20 μL OSs per larva. Secretions were immediately transferred to pre-cooled 1.5 mL microcentrifuge tubes on ice. Pooled samples were centrifuged at 12 000 rpm for 10 min at 4 °C to pellet food debris, and the clarified OS was diluted five-fold with ddH_2_O before use.

Approximately 20 μL of diluted OS secretion was applied per treated leaf. The leaves were collected at 0, 1, 3, 6, 12, and 24 h after volatile exposure for RNA extraction and hormone quantification.

Wild-type plants (cv. Castlemart) were pre-exposed to a β-ocimene mixture or solvent control. Each treatment included three containers, and each exposure container held four plants. After 24 h of β-ocimene exposure, tomato leaves were mechanically wounded with a fabric pattern wheel, and 20 μL of 5-fold diluted *S. litura* oral secretions was immediately applied to the wound sites. Leaf samples were collected at 0, 1, 3, 6, 12, and 24 h post-WOS treatment for subsequent RNA extraction and endogenous hormone quantification. The dynamic accumulation of JA and its bioactive conjugate jasmonoyl-L-isoleucine (JA-Ile), as well as the transcript levels of JA biosynthetic genes *LOXD* and *AOS*, were quantified at multiple time points post-WOS treatment (Figure 6A–D).

To elucidate the molecular mechanism underlying β-ocimene-mediated priming, the JA biosynthesis-deficient tomato mutant *spr8* (carrying a *SlLOXD* loss-of-function mutation) and its wild-type background cultivar Castlemart (CM) were used for genetic verification [26]. Each genotype was exposed to the β-ocimene isomer mixture or solvent control in the container for 24 h. Each exposure container held four plants and each treatment included three containers. Three third-instar *S. litura* larvae were placed on each plant. Weight gain of *S. litura* larvae fed on tomato plants of wild-type CM and JA-deficient mutant *spr8* exposed to β-ocimene was quantified after 24 h (Figure 6E).

### 4.7. RNA Extraction and Real-Time Quantitative PCR (qRT-PCR) Analysis

Total RNA extraction and reverse transcription were performed based on the method of Lin et al. [44] with minor modifications. Briefly, 100 mg of fresh tomato leaf tissue was frozen and homogenized into fine powder with liquid nitrogen. Total RNA was isolated using the Eastep^®^ Super Total RNA Extraction Kit (Promega Biotech Co., Ltd., Beijing, China) following the manufacturer’s standard protocol. Genomic DNA contamination was eliminated by RNase-Free DNase I (TIANGEN Biotech Co., Ltd., Beijing, China) treatment.

Purified total RNA was reverse-transcribed into complementary DNA (cDNA) using the GoScript™ Reverse Transcription System (Promega Biotech Co., Ltd., Beijing, China). The qRT-PCR assay was conducted using the MonAmp™ ChemoHS qPCR Mix (High Rox) Kit (Monad Biotech Co., Ltd., Suzhou, China). The thermal cycling program was set as: 95 °C for 5 min, followed by 40 cycles of 95 °C for 10 s, 56 °C for 10 s, and 72 °C for 30 s. Fluorescence signals were detected at the 72 °C extension stage.

The tomato *Actin* gene was used as the internal reference gene. Relative gene expression levels were calculated using the 2^−ΔΔCT^ method. All gene-specific primers used in this study are listed in Table S1. Three independent biological replicates were performed for each sample group.

### 4.8. Quantification of Endogenous Plant Hormones

Fresh tomato leaf samples were harvested, weighed, immediately snap-frozen in liquid nitrogen, and stored at −80 °C until hormone extraction. For hormone quantification, frozen leaf powder (50 mg) was homogenized in a precooled extraction solution consisting of methanol, water, and formic acid (15:4:1, *V*/*V*/*V*). The homogenized extracts were dried completely under nitrogen gas, redissolved in 80% (*V*/*V*) methanol solution, and filtered through a 0.22 μm PTFE membrane (Anpel) prior to detection.

Endogenous concentrations of jasmonic acid (JA) and jasmonoyl-L-isoleucine (JA-Ile) were quantified via liquid chromatography–tandem mass spectrometry (LC-MS/MS) following the established protocol described by Šimura et al. [45]. All hormone quantification experiments were performed with three independent biological replicates.

### 4.9. Statistical Analysis

All raw data were sorted and preliminarily processed using Microsoft Excel 2019. Graph plotting was performed with GraphPad Prism 9. Statistical significance was determined using SPSS 24.0 software. All experiments were designed with a completely randomized layout. The pairwise comparisons displayed in the figures used two-sided Student’s *t*-tests, with exact *p* values reported in the Section 2 where available. For the CM/*spr8* bioassay, genotype, exposure, and genotype × exposure terms were assessed by two-way analysis of variance. Larvae fed on the same plant were treated as subsamples because they shared the same plant phenotype and exposure history, and plants exposed within one container shared a common exposure unit. Larval numbers (*n* value) in the figures denote the numbers of surviving larvae unless otherwise stated, not the numbers of independent exposure replicates.

## 5. Conclusions

This study demonstrates that insect herbivory by *S. litura* induces multiple tomato TPS genes, and controlled pre-exposure to β-ocimene enhances antiherbivore resistance in intact tomato plants. β-Ocimene-triggered antiherbivore defense is dependent on endogenous JA accumulation in tomato. These findings enrich our understanding of the ecological functions and molecular mechanisms of β-ocimene in plant–insect interactions.

## Data Availability

The original contributions presented in this study are included in the article/Supplementary Materials. Further inquiries can be directed to the corresponding authors.

## Supplementary Materials

The following supporting information can be downloaded at https://www.mdpi.com/article/10.3390/plants15193049/s1: Figure S1: Experimental design for pre-exposure to β-ocimene to antiherbivore defense in tomato plants; Table S1: qRT-PCR primers for qRT-PCR.
