# Supplementary material for: Pre-Exposure to Volatile β-Ocimene Enhances Tomato Antiherbivore Resistance Against Polyphagous Lepidopteran Pest Spodoptera litura (Fabricius)
Source: Plants (Basel). 2026 Oct 6;15(19):3049. doi: 10.3390/plants15193049 (PMC13667161; doi:10.3390/plants15193049)
Supplement: Supplementary file 1 [file plants-15-03049-s001.zip › plants-4497067-supplementary.pdf]

Supplementary Materials

Article title: **Pre-Exposure to Volatile β-Ocimene Enhances Tomato Antiherbivore Resistance Against Polyphagous Lepidopteran Pest Spodoptera litura (Fabricius)**

Supporting Information:

Table S1

Figure S1

Table S1 qRT-PCR primers for qRT-PCR

Gene name	Primer sequences
GGPPS3	F: 5'- GGGATGCACTTCTTGCATTAG -3'
	R: 5'- CTACAACCTGACCAGCTACAA -3'
SSU I	F: 5'- GGACAGCTAGAAGGCCAATATC -3'
	R: 5'- GCTCCACATGCATGAATTTCC -3'
SSU II	F: 5'- TCGAGTCCTCCGGGTTATTA -3'
	R: 5'- CACTCACCCATTTCACCATATTTC -3'
FPPS1	F: 5'- TCTTCGCAACCACATCACCA -3'
	R: 5'- CCAGAGGCAGTCTGGAACTC -3'
TPS3	F: 5'- ATTCGTTGGCCTGCTACGAT -3'
	R: 5'- CCTCTTCTTCACTTGCCCCA -3'
TPS4	F: 5'- TCACTCTTGCTGTTCAAAGATGG -3'
	R: 5'- TGAGAACCTCATACGCCACG -3'
TPS5	F: 5'- ACAGTTCCAAGGACGAGCAT -3'
	R: 5'- GTGCCAAAGTGGATTTTGTGGT -3'
TPS7	F: 5'- ACACGCTGAAAAGTGCAACA -3'
	R: 5'- AGTTCCAAAGCATGGACCACT -3'
TPS8	F: 5'- TGGACGATATGTCTTCCCACT -3'
	R: 5'- GGCACAAGTCCCATATCGATTC -3'
TPS9	F: 5'- ATCCAGAGATGGGATGCTAATG -3'
	R: 5'- CACGGTCCAGTTTACCTTCTT -3'
TPS25	F: 5'- ACCGTTGCCATAGAGAAGTGG -3'
	R: 5'- GCTTTACAGAAGTTGACCCACAC -3'
TPS27	F: 5'- CCACTGCATTGGAAGGTGGA -3'
	R: 5'- TCTGATGCTCAGCTTGAACCA -3'
TPS31	F: 5'- GAGATTGTTCGCCCTGTTGC -3'
	R: 5'- CAGCCAATGTTTTTCCTGACAT -3'
TPS32	F: 5'- ATGGCCCTACTTAACAACCAAGA -3'

	R: 5’- TTCCACAGGCAGCATCAGAC -3’
TPS36	F: 5’- AAGATCTGGACCTGGCAAGC -3’
	R: 5’- TTGCGCGACTATACTGAGGC -3’
TPS38	F: 5’- GAAGCAGTGGGCTAACCTGT -3’
	R: 5’- GTACAGGACCGGTGTTGGTT -3’
OPR3	F: 5’-ATGTTGGTCGTGCATCTCAT-3’
	R:5’-GGTTCCAATTGCTCTTGGTT-3’
Actin	F: 5'-TGGTCGGAATGGGACAGAAG-3'
	R: 5'-CTCAGTCAGGAGAACAGGGT-3'

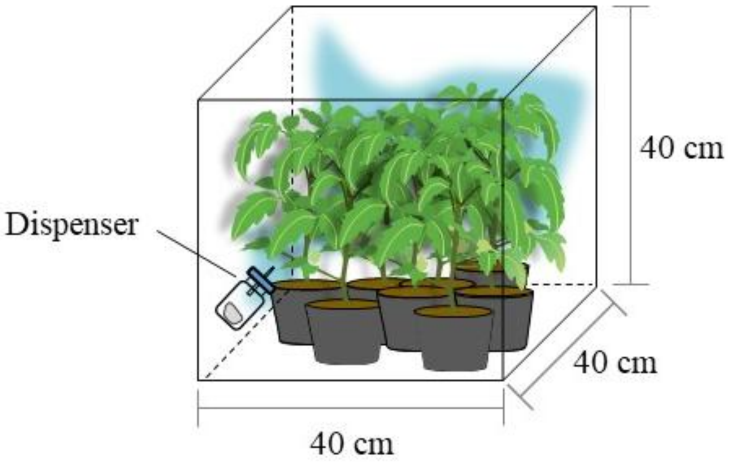

Figure S1 Experimental design for pre-exposure to β-ocimene to antiherbivore defense in tomato plants.
